# The effectiveness of 8A model death education on the reduction of death depression: A preliminary study

**DOI:** 10.1002/nop2.390

**Published:** 2019-10-10

**Authors:** Mahboubeh Dadfar, David Lester

**Affiliations:** ^1^ School of Behavioral Sciences and Mental Health‐Tehran Institute of Psychiatry International Campus School of Public Health, Student Committee of Education and Development Center (EDC) Spiritual Health Research Center Iran University of Medical Sciences Tehran Iran; ^2^ Stockton University Galloway NJ USA

**Keywords:** 8A model, death depression, death education, Iran, nurses

## Abstract

**Aim:**

Death education using the 8A model can reduce death distress and promote mental health. The aim of the present study was to investigate the effectiveness of the 8A model death education programme for reducing death depression among nurses. The hypothesis was that participating in the 8A model death education programme would reduce death depression.

**Design:**

A pre‐test–post‐test intervention.

**Methods:**

Ten nurses were selected randomly from the intensive care units and critical care units wards of the Khatom‐Al‐Anbia General Hospital in Tehran, Iran. They completed the Death Depression Scale before and after intervention. The 8A model was conducted in six workshops weekly, each of 6 hr, for a total of 36 hr.

**Results:**

There was a significant difference between pre‐test and post‐test on the Death Depression Scale scores.

**Discussion:**

The 8A model appears to be useful for the reduction of death depression and the promotion of mental health in the sample. However, the model should be tested on larger samples and with a control group before concluding that the model is effective in reducing death distress.

## 1 BACKGROUND

Death depression, the second component of reactions to death introduced by Templer, Lavoie, Chalgujian, and Thomas‐Dobson (1990), is an emotional, attitude and cognitive construct. The rationale for introducing this construct was that depression appeared to be present in the anticipation of death and in the process of dying. Death depression (feelings of despair, loneliness, dread and a type of sadness) can also be a reaction to the death of a close friend or family member and as one's own death. Erikson (1982) stated that, in the last psychosocial stage of life, individuals who lack a coherent sense of self consider themselves to be failures and experience despair. In the theory of Kübler‐Ross (1969), there are five stages of grief, death and the dying process in terminal illness, summarized as DABDA: Denial, Anger, Bargaining, Depression and Acceptance. In the Depression stage, people become depressed over their death and may become silent, refuse visitors and spend much of the time sullen and in mourning (Afonso, & Minayo, 2013; Burnier, 2017; Kübler‐Ross, & Kessler, 2014). In the Depression stage of the grief process, common signs of depression are difficulty sleeping, poor appetite, fatigue, lack of energy, crying spells, self‐pity, and feeling lonely, isolated, empty, lost and anxious (Dadfar, & Lester, 2017a; Dadfar, Lester, Asgharnejad Farid, Atef Vahid, & Birashk, 2014; Tomás‐Sábado & Gómez‐Benito, 2005; Tomás‐Sábado & Limonero, 2007).

## 2 PROBLEM IDENTIFICATION

Depressive symptoms are associated with, or exacerbated by, existential despair and a lack of meaning in life (Ghaemi, 2007; Havens & Chaemi, 2005). These symptoms have been reported also in health professionals who work with dying patients (Tomas–Sabado, Limonero, Templer, & Gómez‐Benito, 2005). Depression may be associated with less buffering against death concerns. Research has shown that mildly depressed individuals demonstrate a greater worldview defence in response to reminders of death compared with non‐depressed individuals (Simon, Greenberg, Harmon‐Jones, Solomon, Greenberg, Harmon‐Jones, Solomon, & Pyzzczynsid, 1996).

Death depression is associated with death distress and death anxiety (Chibnall, Videen, Duckro, & Miller, 2002; Lester, 2003). Significant correlations have been reported between death depression and death anxiety (Almostadi, 2012; Dadfar, Abdel‐Khalek, & Lester, 2017, 2018; Dadfar, & Lester, 2016, 2017a, 2017b, 2018; Dadfar, Lester, & Abdel‐Khalek, 2018; Tomás‐Sábado & Gómez‐Benito, 2005), also between death depression, death anxiety, death obsession and reasons for fearing death (Abdel‐Khalek, 1998, 2002, 2004, 2012; Abdel‐Khalek, Dadfar, & Lester, in submission; Ayyad, 2013; Dadfar, Abdel‐Khalek, & Lester, 2018; Dadfar, & Lester, 2017a; Dadfar et al., 2014).

Religious belief systems play an important role in death concerns, but pre‐occupation with death can cause depression and anxiety even in some religious people (Dadfar, Abdel‐Khalek, Lester, & Atef Vahid, 2017; Dadfar & Bahrami, 2016; Dadfar, Bahrami, Sheybani Noghabi, & Askari, 2016; Dadfar, & Lester, 2017b; Dadfar, Lester, Abdel‐Khalek, & Ron, 2018; Dadfar, Lester, & Bahrami, 2016). Using Death Depression Scale (DDS), Alvarado, Templer Bresler and Thomas‐Dobson (1995) reported that lower death depression scores were associated with more confidence in religion and stronger afterlife beliefs. Harville, Stokes, Templer, and Rienze (2004) found that more certainty about life after death was associated with less death depression. Afterlife beliefs played a greater role in lowering DDS scores than a belief in God.

There has been research comparing religiosity and mental health in Muslim and non‐Muslim individuals. For example, Abdel‐Khalek and Lester (2018) found that Egyptian (mainly Muslim students) had higher religiosity scores than did British (mainly Christian) students but lower subjective well‐being, while Abdel‐Khalek and Lester (2009) found that Kuwaiti students had significantly higher fears of death than did American students. There has also been some research on death concerns among Iranian Muslim nurses (See Aghajani, Valiee, & Tol, 2010; Bagherian, Iranmanesh, Dargahi, & Abbaszadeh, 2010; Dadfar, Asgharnejad Farid, Atef Vahid, Lester, et al., 2014; Dadfar, & Lester, 2014a, 2014b, 2015; Dadfar, Lester, Atef Vahid, Asgharnejad Farid, & Birashk, 2015; Naderi, Bakhtiar Poor, & Shokouhi, 2010; Rajabi, Begdeli, & Naderi, 2015). The 8A model is based on the transTtheoretical model (TTM) introduced by Chan, Tin, Chan, and Chan (2010) for the Empowerment Network of Adjustment to Bereavement and Loss in End‐of‐life (ENABLE) programs. The model is one of the newest approaches to death education (See also Chan et al., 2010; Dadfar, Asgharnejad Farid, Lester, Atef Vahid, & Birashk, 2016; Dadfar, Lester, Asgharnejad Farid, Atef Vahid, & Birashk, 2017).

The aim of the present study was to investigate the effectiveness of the 8A model death education programme for reducing death depression among nurses. The research question was that there will be a difference in death depression in nurses who participated in 8A model death education comparing pre‐test to post‐test. The hypothesis was that participation in the 8A model death education programme would reduce death depression from pre‐test to post‐test.

## 3 METHODS

The participants were 10 nurses selected randomly from the intensive care units (ICU) and critical care units (CCU) wards of the Khatom‐Al‐Anbia General Hospital in Tehran, Iran. The nurses completed the Death Depression Scale (DDS) before and after the intervention. The 8A model programme was conducted in six workshops weekly of 6 hr each for a total of 36 hr. Inclusion criteria were as follows: nurses working in the two types of wards and an educational level of bachelor's degree or higher. Exclusion criteria were the following: having medical diseases and mental disorders and receiving individual or group psychoeducational and psychological interventions.

The nurses’ participation in the study was voluntary and anonymity was ensured. The objective of the study was explained to the nurses, and they were given certifications that they had completed the programme.

### 3.1 Measure

#### 3.1.1 The Death Depression Scale (DDS)

The DDS was devised by Templer et al. (1990). The DDS is a 17‐item self‐report questionnaire and consists of six elements: death despair (items 8, 11 and 16), death loneliness (items 4, 9, 10 and 13), death dread/fear (items 14, 15 and 16), death sadness (items 2 and 3), death depression (items 2 and 12) and death finality/end (items 6 and 7). The DDS has two different formats: a true/false or yes/no format and a five‐point Likert format. The present study used the true/false format. Two items (items of 11 and 12) of 17 items the DDS control for an acquiescence response set. Total scores can range from 0 to 17. Higher scores on the DDS indicate more death depression (Fischer & Corcoran, 2007). Several studies have demonstrated that the DDS has good internal consistency, validity and reliability (Abdel‐Khalek, 1998, 2004; Aghazadeh, Mohammadzadeh, & Rezaie, 2014; Almostadi, 2012; Alvarado, Templer, Bresler, & Thomas‐Dobson, 1993; Dadfar, & Lester, 2017a; Dadfar, & Lester, in press; Mohammadzadeh, Rezaei, & Aghazadeh, 2016; Rajabi et al., 2015; Sharif Nia et al., 2017; Templer et al., 1990, 2002 ; Tomás‐Sábado & Gómez‐Benito, 2005; Tomás‐Sábado & Limonero, 2007; Tomas–Sabado et al., 2005).

### 3.2 Data analysis

For determination of normality of the data and equality of variances, Kolmogorov–Smirnov test and Levene's test were used, respectively. The data were analysed with descriptive statistics (mean and standard deviations) and inferential statistics (paired/dependent *t* test) using the SPSS/WIN 16.0 program.

## 4 FINDINGS

The mean age of the nurses was 36.6 (*SD* = 11.8). Mean work experience was 13.7 years (*SD* = 10.7). About 90% of nurses were women. About 70% married, and 80% had a BA degree. The appointment was contracted for hours per week or permanent (50% each of them). Table 1 gives some demographic and professional data on the sample.

**Table 1:** Characteristics of the sample

Variable	*N*	%
Position		
Staff nurse	8	80.0
Head nurse	2	20.0
Work shift		
Rotational	4	30.0
Fixed	6	70.0
The number of patients per shift		
0–9	9	90.0
Care of end‐stage patients in the past 3 months		
0–6	8	80.0
Participation in resuscitation operations in the past 3 months		
>5	3	30.0

There was a significant difference between pre‐test and post‐test scores for the DDS scores. The nurses’ mean score decreased from 8.70 (*SD* = 5.96) to 4.80 (*SD* = 4.21) (t = 2.38, *df* = 9, *p* = .04).

## 5 DISCUSSION

The aim of the present study was to investigate the effectiveness of the 8A model death education on the reduction of death depression among nurses. The results indicated a significant difference between pre‐test and post‐test for the DDS scores. Consistent with these results, the effectiveness of death education different programmes on attitudes to death has been reported in other research on healthcare professionals (Chan et al., 2010; Dadfar et al., 2016, 2017, 2015; Dadfar, & Lester, 2014a; Dadfar, Lester, Birashk, Asgharnejad Farid, & Atef Vahid, 2016).

Menzies, Zuccala, Sharpe, and Dar‐Nimrod (2018) conducted a meta‐analysis of 15 studies on reducing death anxiety. Some involved education (including lectures, readings and discussion), while others were more psychotherapeutically based (using, e.g., cognitive behavioural therapy), but none of the studies reviewed in this meta‐analysis compared different models. The 8A model is educationally based rather than psychotherapeutically based, and future research should explore the relative impact of different models in order to see which are more effective.

The limitations of the present study include the relatively small sample of nurses who were mostly female (90%); the sample was restricted to one hospital, limiting the representativeness of the findings; there was no control group; and the training had no follow‐up phase. Future research should address these issues and addressing whether and in which way these programmes need to be modified taking into account the culture (religion, educational level, gender, etc.) of the participants. It would also be of interest to explore in detail how having had a death education programme changes the particular behaviours of nurses towards their patients, perhaps using participant observation research.

The results of the present study do have important implications. Since nurses have to cope with dying patients in the course of their work, healthcare administrators should provide programmes to minimize death anxiety as part of the regular continued training for nurses and other healthcare workers. Because educational programmes, such as the 8A model, appear to be effective, this type of programme may be preferable for nurses by being less intrusive than psychotherapy‐based programmes which may imply that the problem lies within the psyche of the nurses. These programmes may need to be extended to all staff working in hospitals, palliative care settings and hospices, such as secretaries and janitors. All types of staff can come into contact with dying patients and so could benefit from these programmes. Providing debriefing sessions after a traumatic incident is another way to combat death anxiety. Organizations should conduct small, weekly group meetings for staff members who experience traumatic situations in order to debrief them.

## 6 CONCLUSION

We conclude that the 8A model was useful for the reduction of death depression and promotion of mental health in this sample of nurses. However, the model should be tested on larger samples and with a control group before concluding that the model is effective in reducing death distress.

## CONFLICT OF INTEREST

The authors declare that there is no funding source, and they have no conflict of interest about the publication of this paper.

## AUTHOR CONTRIBUTIONS

All authors have agreed on the final version and met at least one of the following criteria [recommended by the ICMJE (http://www.icmje.org/recommendations/)]: substantial contributions to conception and design, acquisition of data or analysis and interpretation of data; drafting the article or revising it critically for important intellectual content.

## ETHICAL APPROVAL

This paper is based on a doctoral thesis in clinical psychology by the senior author. Research Ethics Committee approval of Iran University of Medical Sciences authorized the permission to conduct this study.

## References

[nop2390-bib-0001] Abdel‐Khalek, A. M. (1998). Death, anxiety, and depression in Lebanese under graduates. OMEGA: Journal of Death and Dying, 37(4), 289–302. 10.2190/CN5K-XF4C-2NPG-17E0

[nop2390-bib-0002] Abdel‐Khalek, A. M. (2002). Death, anxiety, and depression: A comparison between Egyptian, Kuwaiti, and Lebanese undergraduates. OMEGA: Journal of Death and Dying, 45(3), 277–287. 10.2190/CNP8-BN0U-HB5X-13TG

[nop2390-bib-0003] Abdel‐Khalek, A. M. (2004). A general factor of death distress in seven clinical and non‐clinical groups. Death Studies, 28(9), 889–898. 10.1080/07481180490491040 15493083

[nop2390-bib-0004] Abdel‐Khalek, A. M. (2012). The death distress construct and scale. OMEGA: Journal of Death and Dying, 64(2), 171–184. 10.2190/OM.64.2.e 22375351

[nop2390-bib-0005] Abdel‐Khalek, A. M. , Dadfar, M. , & Lester, D. (In submission). Death depression in Egyptian clinical and non‐clinical groups. Death Studies. 10.1002/nop2.601 PMC7729647 33318811

[nop2390-bib-0006] Abdel‐Khalek, A. M. , & Lester, D. (2009). Death anxiety as related to somatic symptoms in two cultures. Psychological Reports, 105, 409–410. 10.2466/PR0.105.2.409-410 19928602

[nop2390-bib-0007] Abdel‐Khalek, A. M. , & Lester, D. (2018). Subjective well‐being and religiosity: Significant associations among college students from Egypt and the United Kingdom. International Journal of Culture & Mental Health, 11, 332–337. 10.1080/17542863.2017.1381132

[nop2390-bib-0008] Afonso, S. B. , & Minayo, M. C. (2013). A reappraisal of the works of Elisabeth Kubler‐Ross. Ciência & Saúde Coletiva, 18(9), 2729–2732. 23989580 10.1590/s1413-81232013000900028

[nop2390-bib-0009] Aghajani, M. , Valiee, S. , & Tol, A. (2010). Death anxiety amongst nurses in critical care and general wards. Iranian Journal of Nursing, 23(67), 59–68.

[nop2390-bib-0010] Aghazadeh, S. E. , Mohammadzadeh, A. , & Rezaie, A. (2014). Validation of Death Depression Scale (DDS) in university students in 2012. Journal of Research & Behavioral Sciences, 12(3), 433–442.

[nop2390-bib-0011] Almostadi, D. A. (2012). The relationship between death depression and death anxiety among cancer patients in Saudi Arabia. Graduate Dissertation. University of sought Florida, USA.

[nop2390-bib-0012] Alvarado, K. A. , Templer, D. I. , Bresler, C. , & Thomas‐Dobson, S. (1993). Are death anxiety and death depression distinct entities? OMEGA: Journal of Death and Dying, 26(2), 113–118. 10.2190/20HL-33JR-VABJ-DLTW

[nop2390-bib-0013] Alvarado, K. A. , Templer, D. I. , Bresler, C. , & Thomas‐Dobson, S. (1995). The relationship of religious variables to death depression and death anxiety. Journal of Clinical Psychology, 51(2), 202–204. 10.1002/1097-4679(199503)51:2<202:AID-JCLP2270510209>3.0.CO;2-M 7797643

[nop2390-bib-0014] Ayyad, F. (2013). Death distress among two samples of lower and higher stress in health care professionals. Psychological Reports, 113(1), 1332–1341. 10.2466/16.09.PR0.113x10z4 24340820

[nop2390-bib-0015] Bagherian, S. , Iranmanesh, S. , Dargahi, H. , & Abbaszadeh, A. (2010). The attitude of nursing staff of institute cancer and Valie‐Asr hospital toward caring for dying patients. Journal of Razi Nursing Midwifery College in Kerman, 9(1 & 2), 8–14.

[nop2390-bib-0016] Burnier, D. (2017). A battle of words: "dignity" and "peace" in the writings of Elisabeth Kübler‐Ross. Journal of Palliative Care, 32(2), 49–54. 10.1177/0825859717717154 28707554

[nop2390-bib-0017] Chan, W. C. H. , Tin, A. F. , Chan, C. H. Y. , & Chan, C. L. W. (2010). Introducing a model 8A in death education training: Promoting planning for end‐of‐life care for Hong Kong Chinese. Illness, Crisis and Loss, 18(1), 49–62.

[nop2390-bib-0018] Chibnall, J. T. , Videen, S. D. , Duckro, P. N. , & Miller, D. K. (2002). Psychosocial‐spiritual correlates of death distress in patients with life‐threatening medical conditions. Palliative Medicine, 16(4), 331–338. 10.1191/0269216302pm544oa 12132546

[nop2390-bib-0019] Dadfar, M. , Abdel‐Khalek, A. M. , & Lester, D. (2017). Psychometric characteristics of the Reasons for Death Fear Scale with Iranian nurses. International Journal of Nursing Sciences, 4(4), 384–388. 31406782 10.1016/j.ijnss.2017.10.002 PMC6626188

[nop2390-bib-0020] Dadfar, M. , Abdel‐Khalek, A. M. , & Lester, D. (2018). Validation of the Farsi version of Death Obsession Scale with nurses. International Journal of Nursing Sciences, 5(2), 186–192. 31406823 10.1016/j.ijnss.2018.04.004 PMC6626241

[nop2390-bib-0021] Dadfar, M. , Abdel‐Khalek, A. M. , Lester, D. , & Atef Vahid, M. K. (2017). The psychometric parameters of the Farsi form of the Arabic Scale of Death Anxiety. The Scientific World Journal, 2017, 7468217, 8 pages. 10.1155/2017/7468217 28698887 PMC5494095

[nop2390-bib-0022] Dadfar, M. , Asgharnejad Farid, A. A. , Atef Vahid, M. K. , Lester, D. , & Birashk, B. (2014). Reasons for fearing death in Iranian nurses. Global Journal on Advances in Pure & Applied Sciences, 4, 335–341.

[nop2390-bib-0023] Dadfar, M. , Asgharnejad Farid, A. A. , Lester, D. , Atef Vahid, M. K. , & Birashk, B. (2016). Effectiveness of death education program by methods of didactic, experimental, and 8A model on the reduction of death distress among nurses. International Journal of Medical Research & Health Sciences, 5(7), 60–71.

[nop2390-bib-0024] Dadfar, M. , & Bahrami, F. (2016). Reliability and factor structure of the Farsi version of the Arabic Scale of Death Anxiety in an Iranian middle‐aged sample. The Scientific World Journal, 2016, 9457041, 5 pages. 28004035 10.1155/2016/9457041 PMC5149686

[nop2390-bib-0025] Dadfar, M. , Bahrami, F. , Sheybani Noghabi, F. , & Askari, M. (2016). Relationship between religious spiritual well‐being and death anxiety in Iranian elders. International Journal of Medical Research & Health Sciences, 5(6), 283–287.

[nop2390-bib-0026] Dadfar, M. , & Lester, D. (in press). The death depression scale: Description and applications, In Preedy V. R. et al. (Eds.), The Neuroscience of depression. Elsevier publishing.

[nop2390-bib-0027] Dadfar, M. , & Lester, D. (2014a). Death education program: A practical guide for healthcare professionals. Tehran, Iran: Mir‐Mah Publications.

[nop2390-bib-0028] Dadfar, M. , & Lester, D. (2014b). Fear of death in Iranian nurses. Shefaye Khatam, 2(S1), 86.

[nop2390-bib-0029] Dadfar, M. , & Lester, D. (2015). Death concern and death obsession in Iranian nurses. Psychological Reports, 116(3), 704–709. 10.2466/12.13.PR0.116k30w5 26030202

[nop2390-bib-0030] Dadfar, M. , & Lester, D. (2016). The reliability, validity, and factorial structure of the Collett‐Lester Fear of Death Scale in a sample of Iranian nurses. International Journal of Medical Research & Health Sciences, 5(Issue 7S), 306–317.

[nop2390-bib-0031] Dadfar, M. , & Lester, D. (2017a). Cronbach's α reliability, concurrent validity, and factorial structure of the Death Depression Scale in an Iranian hospital staff sample. International Journal of Nursing Sciences, 4(2), 135–141. 10.1016/j.ijnss.2017.02.007 31406733 PMC6626102

[nop2390-bib-0032] Dadfar, M. , & Lester, D. (2017b). Religiosity, spirituality and death anxiety. Austin Journal of Psychiatry and Behavioral Sciences, 4(1), 1061

[nop2390-bib-0033] Dadfar, M. , & Lester, D. (2018). The Farsi translation, reliability and validity of the Death Concern Scale. Trends in Psychiatry and Psychotherapy, 40(2), 114–125. 10.1590/2237-6089-2017-0078 29995157

[nop2390-bib-0034] Dadfar, M. , Lester, D. , & Abdel‐Khalek, A. M. (2018). Validity and reliability of the Farsi version of the Death Anxiety Scale with nurses. Illness, Crisis & Loss, First Published November 14, 2018. 10.1177/1054137318810232

[nop2390-bib-0035] Dadfar, M. , Lester, D. , Abdel‐Khalek, A. M. , & Ron, P. (2018). Death anxiety in Muslim Iranians: the comparison between youths, middle adults and late adults. Illness, Crisis & Loss, First Published August 19, 2018. 10.1177/1054137318790080

[nop2390-bib-0036] Dadfar, M. , Lester, D. , Asgharnejad Farid, A. A. , Atef Vahid, M. K. , & Birashk, B. (2014). Death depression in Iranian nurses. Advances in Environmental Biology, 8(13), 218–222.

[nop2390-bib-0037] Dadfar, M. , Lester, D. , Asgharnejad Farid, A. A. , Atef Vahid, M. K. , & Birashk, B. (2017). 8A conceptual model for death education. Shefaye Khatam, 5(4), 98–109. 10.18869/acadpub.shefa.5.4.98

[nop2390-bib-0038] Dadfar, M. , Lester, D. , Atef Vahid, M. K. , Asgharnejad Farid, A. A. , & Birashk, B. (2015). Death distress in nurses: Psychoeducational interventions. Tehran, Iran: Mir‐Mah Publications.

[nop2390-bib-0039] Dadfar, M. , Lester, D. , & Bahrami, F. (2016). Death anxiety, reliability, validity, and factorial structure of the Farsi Form of the Arabic Scale of Death Anxiety in Iranian old‐aged individuals. Journal of Aging Research, 2016, 2906857, 7 pages. 27867662 10.1155/2016/2906857 PMC5102731

[nop2390-bib-0040] Dadfar, M. , Lester, D. , Birashk, B. , Asgharnejad Farid, A. A. , & Atef Vahid, M. K. (2016). The effectiveness of didactic approach on the reduction of death obsession. Shefaye Khatam, 4(S1), 29.

[nop2390-bib-0041] Erikson, E. H. (1982). The life cycle completed. New York, NY: Norton.

[nop2390-bib-0042] Fischer, K. , & Corcoran, J. (2007). Measures for clinical practice and research: A sourcebook two‐volume set. (4th ed.). Oxford, England: Oxford University Press.

[nop2390-bib-0043] Ghaemi, S. N. (2007). Feeling and time: The phenomenology of mood disorders, depressive realism, and existential psychotherapy. Schizophrenia Bulletin, 33, 122–130. 10.1093/schbul/sbl061 17122410 PMC2632297

[nop2390-bib-0044] Harville, M. , Stokes, S. J. , Templer, D. I. , & Rienzi, B. (2004). Relation of existential and religious variables to the Death Depression. Scale‐Revised. OMEGA: Journal of Death and Dying, 48(2), 165–184. 10.2190/C2MJ-5DXN-EU6C-N2XR

[nop2390-bib-0045] Havens, L. L. , & Chaemi, S. N. (2005). Existential despair and bipolar disorder: The therapeutic alliance as a mood stabilizer. American Journal of Psychotherapy, 59, 137–147. 10.1176/appi.psychotherapy.2005.59.2.137 16170918

[nop2390-bib-0046] Kübler‐Ross, E. (1969). On death and dying. New York, NY: Routledge.

[nop2390-bib-0047] Kübler‐Ross, E. , & Kessler, D. (2014). On grief & grieving: Finding the meaning of grief through the five stages of loss. New York, NY: Scribner.

[nop2390-bib-0048] Lester, D. (2003). Death anxiety, death depression and death obsession. Psychological Reports, 93(3 Pt 1), 695–696. 10.2466/PR0.93.7.695-696 14723430

[nop2390-bib-0049] Menzies, R. E. , Zuccala, M. , Sharpe, L. , & Dar‐Nimrod, I. (2018). The effects of psychosocial interventions on death anxiety: A meta‐analysis and systematic review of randomized controlled trials. Journal of Anxiety Disorders, 59, 64–73. 30308474 10.1016/j.janxdis.2018.09.004

[nop2390-bib-0050] Mohammadzadeh, A. , Rezaei, A. , & Aghazadeh, S. E. (2016). Validation of likert form death depression scale in a university students samples. Journal of Ilam University of Medical Sciences, 24(1), 89–97.

[nop2390-bib-0051] Naderi, F. , Bakhtiar Poor, S. , & Shokouhi, M. (2010). The comparison of death anxiety, optimism and sense of humor among nurses women. Woman and Culture, 1(3), 41–50.

[nop2390-bib-0052] Rajabi, G. R. , Begdeli, Z. , & Naderi, Z. (2015). Psychometric properties of the Persian version of Death Depression Scale among nurses. Death Studies, 39(6), 342–346. 10.1080/07481187.2014.951495 25849995

[nop2390-bib-0053] Sharif Nia, H. , Pahlevan Sharif, S. , Lehto, R. H. , Allen, K. A. , Goudarzian, A. H. , Yaghoobzadeh, A. , & Soleimani, M. A. (2017). Psychometric evaluation of Persian version of the Death Depression Scale in Iranian patients with acute myocardial Infarction. Iranian Journal of Psychiatry, 12(3), 172–181. 29062368 PMC5640578

[nop2390-bib-0054] Simon, L. , Greenberg, J. , Harmon‐Jones, E. , Solomon, S. , & Pyzzczynsid, T. (1996). Mild depression, mortality salience and defense of the worldview evidence of intensified Terror Management in the mildly depressed. Personality and Social Psychology Bulletin, 22(1), 81–90. 10.1177/0146167296221008

[nop2390-bib-0059] Templer, D. I. , Harville, M. , Hutton, S. , Underwood, R. , Tomeo, M. , Tomeo, M. , Russell, M. , … Ankawa, H. (2002). Death Depression Scale- Revised. OMEGA: Journal of Death and Dying, 44, 105–112. 10.2190/32L3-DPDA-M4U3-7L81

[nop2390-bib-0055] Templer, D. I. , Lavoie, M. , Chalgujian, H. , & Thomas‐Dobson, S. (1990). The measurement of death depression. Journal of Clinical Psychology, 46(6), 834–839. 10.1002/1097-4679(199011)46:6<834:AID-JCLP2270460623>3.0.CO;2-0 2286679

[nop2390-bib-0056] Tomás‐Sábado, J. , & Gómez‐Benito, J. (2005). Death anxiety and death depression in Spanish nurses. Psychological Reports, 97, 21–24. 10.2466/PR0.97.5.21-24 16279299

[nop2390-bib-0057] Tomás‐Sábado, J. , & Limonero, J. T. (2007). Death depression and death obsession: Are they different constructs? Psychological Reports, 100, 755–758. 10.2466/pr0.100.3.755-758 17688089

[nop2390-bib-0058] Tomas‐Sabado, J. , Limonero, J. T. , Templer, D. I. , & Gómez‐Benito, J. (2005). The Death Depression Scale‐Revised. Preliminary empirical validation of the Spanish form. OMEGA: Journal of Death and Dying, 50(1), 43–52. 10.2190/XBRR-PMPP-C0XH-KP80

